# The Association between Telomere Length and Cancer Prognosis: Evidence from a Meta-Analysis

**DOI:** 10.1371/journal.pone.0133174

**Published:** 2015-07-15

**Authors:** Chunli Zhang, Xiaohua Chen, Lu Li, Ying Zhou, Chao Wang, Shuxun Hou

**Affiliations:** 1 Institute of Orthopedics, the First Affiliated Hospital of Chinese People's Liberation Army General Hospital, Beijing, China; 2 Medical School of Chinese People's Liberation Army, Beijing, China; 3 Department of Laboratory Medicine, No 161 Hospital of People's Liberation Army, Wuhan, China; 4 Key Laboratory of Environment and Health, Ministry of Education & Ministry of Environmental Protection, and State Key Laboratory of Environmental Health (Incubating), School of Public Health, Tongji Medical College, Huazhong University of Science and Technology, Wuhan, China; 5 Department of General Surgery, Tongji Hospital, Tongji Medical College, Huazhong University of Science and Technology, Wuhan, China; University of Newcastle, UNITED KINGDOM

## Abstract

**Background:**

Telomeres are essential for chromosomal integrity and stability. Shortened telomere length (TL) has been associated with risk of cancers and aging-related diseases. Several studies have explored associations between TL and cancer prognosis, but the results are conflicting.

**Methods:**

Prospective studies on the relationship between TL and cancer survival were identified by a search of PubMed up to May 25, 2015. There were no restrictions on the cancer type or DNA source. The quality of the included studies was assessed using the Newcastle-Ottawa Scale. Meta-analysis approaches were conducted to determine pooled relative risks and 95% confidence intervals.

**Results:**

Thirty-three articles containing forty-five independent studies were ultimately involved in our meta-analysis, of which twenty-seven were about overall cancer survival and eighteen were about cancer progression. Short TL was associated with increased cancer mortality risk (RR = 1.30, 95%CI: 1.06–1.59) and poor cancer progression (RR = 1.44, 95%CI: 1.10–1.88), both with high levels of heterogeneity (*I^2^* = 83.5%, *P* = 0.012for overall survival and *I^2^* = 75.4%, *P* = 0.008 for progression). TL was an independent predictor of overall cancer survival and progression in chronic lymphocytic leukemia. Besides, short telomeres were also associated with increased colorectal cancer mortality and decreased overall survival of esophageal cancer, but not in other cancers. Cancer progression was associated with TL in Asian and America populations and short TL predicted poor cancer survival in older populations. Compared with tumor tissue cells, TL in blood lymphocyte cells was better for prediction. In addition, the associations remained significant when restricted to studies with adjustments for age, with larger sample sizes, measuring TL using southern blotting or estimating risk effects by hazard ratios.

**Conclusion:**

Short TL demonstrated a significant association with poor cancer survival, suggesting the potential prognostic significance of TL. Additional large well-designed studies are needed to confirm our findings.

## Introduction

Telomeres are specialized structures that adorn the ends of eukaryotic chromosomes. Human telomeres comprise short tandem repeats 5’-TTAGGG-3’ and telomere related proteins [1]. It is generally believed that telomeres protect chromosomes from degradation and end-to-end fusion, thus playing an essential role in maintaining chromosomal integrity [2]. In normal human somatic cells, telomeres ranges from 9 to 15 kb initially, and shrink by about 50–200 nucleotides per replication cycle [3]. With continuous shortening, telomeres reach a critical length and consequently the deficient telomere triggers irreversible cell cycle arrest known as cellular senescence, apoptosis and even malignance [4].

Telomere structure is sensitive to a wide spectrum of endogenous and environmental factors such as aging, oxidative stress, unhealthy lifestyle and genotoxic stress [5]. These factors jointly influence the cell fate jointly by modifying telomere length (TL) and structure and leading to disease occurrence. Therefore, TL has been proposed as an integrated biomarker for age and a general risk for age-associated diseases [6]. In particular, it has been reported that in mice models excessive telomere shortening contributes to the evolution of genome instability and thus is involved in cancer formation [7–9]. Accordingly, telomeres in human tumor cells were usually shorter than those in the surrounding normal tissue cells [10, 11].

Several studies have explored the correlations between TL and the risk of different cancers. Moreover, systematic reviews and meta-analyses have confirmed inverse associations of TL with cancer morbidity and other aging-related diseases [12–15]. There are also some prospective studies which examined the impact of TL on cancer survival, but the results are conflicting. For example, a recent report indicated that patients with shorter leukocyte TL had significantly worse overall survival and relapse-free survival than those with longer TL in colorectal cancer [16]. Conversely, Garcia-Aranda et al. found that long TL predicted poor colorectal cancer prognosis [17]. Svenson et al. showed that longer TL was associated with increased breast cancer death risk [18], while some other studies failed to observe a significant association [19–21]. These apparent discrepancies suggest that individual studies may be under powered for the detection of true associations due to limited sample sizes. Given this reason, we conducted a meta-analysis to provide a comprehensive assessment of the relationship between TL and cancer death and disease progression.

## Methods

### Search strategy

We performed a systematic literature search on PubMed electronic database using the following key words: “telomere length”, “cancer” or “carcinoma” or “tumor”, and “survival” or “prognosis” or “mortality”. The last search was updated on May 25, 2015. To identify more pertinent publications, the reference lists of selected articles were also hand searched.

### Study selection

Studies which met the following criteria were eligible: (1). Study evaluated the relationship between TL and cancer survival with a prospective design. (2). Using long (or longest) TL as reference, the study provided relative risks (RRs) or hazard ratios (HRs) with 95% confidence intervals (CIs) for shorter (or shortest) TL. Alternatively, sufficient information was available to estimate the above effect sizes. (3). Participants must be newly diagnosed cancer patients. Therefore, studies focused on cancer-specific mortality in general population were excluded. (4). Studies analyzing the associations of TL with all-cause mortality were rejected because we were focused on cancer survival. If the same study was reported more than once, we selected the most recently published one to avoid repetition. In addition, if an article presented results for several different cohort studies we considered them as independent studies. There were no restrictions on cancer type, DNA source or measurement of TL.

### Data extraction

For each eligible study, we used a standardized abstraction form to extract the following information: first author, year of publication, mean or middle point of follow-up time, region, cancer type, number of participants, measurement method for TL, DNA source, whether adjusted for age, TL classification (dichotomy, trichotomy or quartile), endpoint (death or disease progression), RRs or HRs with corresponding 95% CIs for short TL versus long TL, and adjusted confounders. If possible, the effect size with the largest degree of adjustment for potential confounders was included.

### Quality assessment

The Newcastle Ottawa Scale (NOS) was used to assess the quality of the included studies [22]. Briefly, each study was judged on three broad dimensions: the selection of the study subjects, the comparability of the study populations and the ascertainment of the outcome of interest in cohort studies. Overall, each study received a total score from zero to nine stars, and a study was considered of high quality if it scored seven or more stars.

### Statistical analysis

To enrich our work, we not only analyzed the association of TL with cancer overall survival, but also extended the outcomes to cancer progression events, including treatment-free survival, relapse-free survival, disease-free survival and progression-free survival. For the vast majority of the studies included in this meta-analysis, a median value (or other cut-point) of the TL in cancer patients divided all subjects into two groups: a short TL group and a long TL group. The association between the TL and cancer survival was examined by RRs or HRs and corresponding 95% CIs with the long telomere as the reference. However, a few studies reported associations for tertile or quartile categories of TL. In these cases, we chose the effect size for shortest telomere versus longest telomere, with the group of longest telomere as the reference. Finally, we combined HRs with RRs and reported RRs as summary effect sizes for simplicity.

The between-study heterogeneity was assessed by the Cochran *Q* test and *I* ^*2*^ statistic and was considered significant if *P* < 0.05 for Cochran *Q* test or *I* ^*2*^ > 50%. When significant heterogeneity was detected, values from different studies were combined using a random-effects model; otherwise, a fixed-effects model was utilized. To evaluate the effect of TL on survival in patients with different cancer types, we detected this relationship by cancer type independently if it was reported in at last two studies. Meta-regression was performed to find the source of heterogeneity. Firstly, an empty regression was run to estimate the baseline value for *tau* ^*2*^. Then, univariate metaregressions were successively conducted with grouping variables. If *tau* ^*2*^ was significantly reduced after one variable entered into the model, then this variable was responsible for heterogeneity among the studies. In subgroup analysis, the included studies were stratified by the following items: age of diagnosis, follow-up time, area, cancer category, whether adjusted for age, number of subjects, technique for TL determination, DNA source, number of divided TL groups and risk type. Sensitivity analyses were performed by excluding each study individually and recalculating the pooled effect sizes. Additionally, we investigated publication bias by funnel plot and Egger’s regression tests.

All analyses were conducted using Stata 10.0. All *P* values were two-sided and a *P* < 0.05 was considered statistically significant.

## Results

### Characteristics of included studies

**Fig 1:**
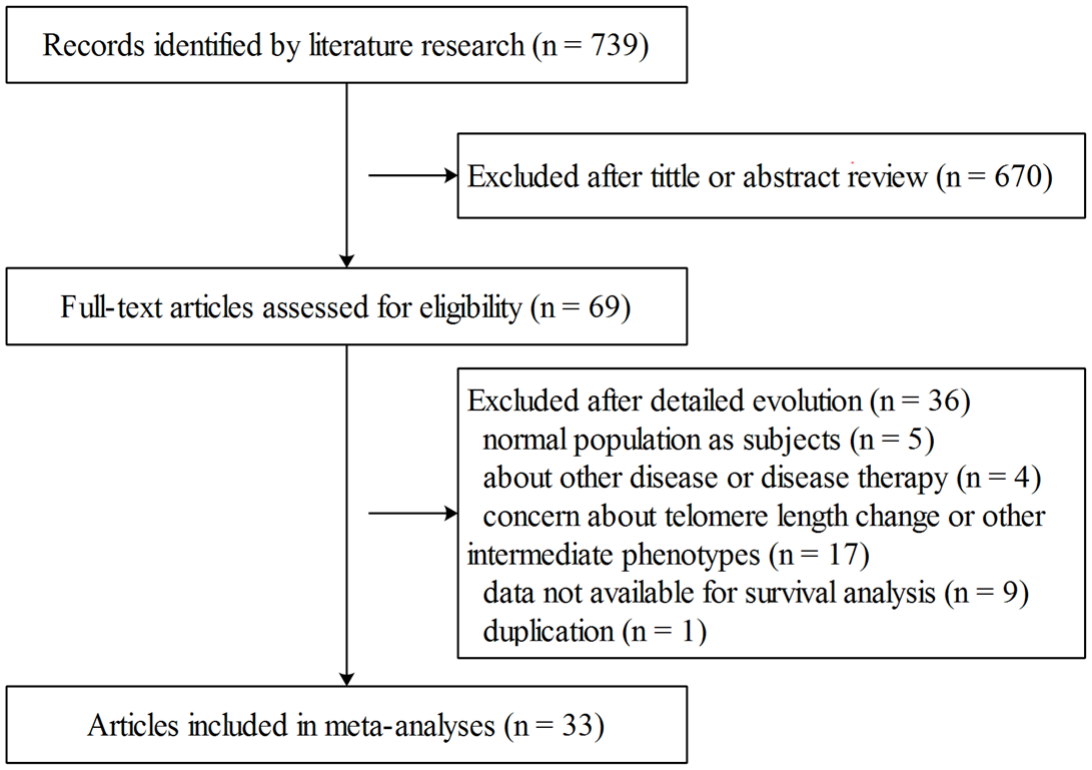
Flow chart for the process of selecting eligible articles.

As shown in Fig 1, the literature search strategy initially identified 739 articles. After scanning the titles and abstracts, sixty-nine publications remained for further investigation. Among these articles, thirty-six were excluded due to the following reasons: normal population as subjects but not cancer patients (n = 5), study on other diseases or disease therapy (n = 4), concerned with TL change or other intermediate phenotypes (n = 17), data not available for survival analysis (n = 9), or duplicated studies (n = 1). There were ten publications which researched both overall survival and disease progression, and one publication collected the results of two independent cohorts, thus they were considered separately. Finally, thirty-three articles containing forty-five independent studies were enrolled in our meta-analysis, of which twenty-seven studies were about cancer overall survival and eighteen studies were about cancer progression [16–21, 23–49]. The essential information for the included studies is listed in Table 1 and S1 Table. Diverse cancers were involved, including chronic lymphocytic leukemia, breast cancer, colorectal cancer, bladder cancer and many others. However, because there were too many cancer types, we combined these cancers into several major categories, including gastrointestinal cancer, female cancer, urinary cancer, neurological cancer, blood cancer and others, in meta-regression and subgroup analysis. The sources of DNA included blood (n = 21), tumor tissue (n = 18), mixed (n = 1) and unclear (n = 1). Quantitative polymerase chain reaction (qPCR) was the primary method for TL determination, while southern blot and fluorescence in situ hybridization-based (FISH) approaches were also utilized. Additionally, European was the predominant ethnic group reported, followed by American and Asian. Thirty-eight out of the forty-five included studies categorized TL into two groups: a long TL group and a short TL group. All the studies except one [44] were of high quality based on our NOS quality assessment.

**Table 1:** Characteristics of included studies.

First author	Year	Cancer	Follow-up time^a^	Region	Method	Sample	TL groups	Risk (95% CI)
Overall survival								
Zhang	2014	ESCC	38 months	Asia	qPCR	tumor tissue	2	0.69 (0.46–1.02)
Duggan	2014	breast cancer	11.2 years	America	qPCR	blood	2	1.32 (0.98–1.79)
Chen	2014	colorectal cancer	28 months	Asia	qPCR	blood	2	2.43 (1.53–3.45)
Russo	2014	bladder cancer	16.3 years	Europe	qPCR	blood	2	3.90 (1.70–9.10)
Weischer	2013	multiple cancers	unclear	Europe	qPCR	blood	4	1.31 (1.14–1.52)
Lötsch	2013	glioblastoma	14.2 months	Europe	qPCR	tumor tissue	2	0.93 (0.57–1.52)
Mansouri	2013	CLL	83 months	Europe	qPCR	blood	2	2.42 (1.40–4.19)
Heaphy	2013	prostate cancer	13.2 years	America	FISH	tumor tissue	2	2.94 (1.35–6.39)
Jeon	2014	NSCLC	unclear	Asia	qPCR	tumor tissue	4	2.43 (1.02–5.79)
Liu	2012	HCC	16.7 months	Asia	qPCR	blood	2	0.49 (0.35–0.68)
Lu	2011	breast cancer	86 months	Europe	qPCR	tumor tissue	2	1.20 (0.71–2.04)
Willeit	2011	multiple cancers	unclear	Europe	qPCR	blood	3	1.52 (1.05–2.21)
Rossi	2009	CLL	32 months	Europe	Southern blot	blood	2	13.31 (3.76–47.05)
Rossi	2009	CLL	54.2 months	Europe	Southern blot	blood	2	1.91 (1.04–3.52)
Svenson	2009	ccRCC	24 months	Europe	qPCR	blood	2	0.33 (0.15–0.77)
Svenson	2008	breast cancer	unclear	Europe	qPCR	blood	2	0.34 (0.16–0.75)
Bechter	1998	CLL	23 months	Europe	Southern blot	tumor tissue	4	3.27 (1.23–8.66)
Kotsopoulos	2014	ovarian cancer	unclear	America	qPCR	blood	2	1.14 (0.91–1.30)
Lin	2014	bladder cancer	21.6 months	America	qPCR	blood	2	1.25 (0.82–1.90)
Shen	2012	breast cancer	8 years	America	qPCR	blood	2	0.87 (0.68–1.13)
Hultdin	2003	CLL	82 months	Europe	Southern blot	combined	2	2.00 (1.21–3.30)
Gertler	2004	colorectal cancer	76 months	Europe	Southern blot	tumor tissue	2	3.56 (1.18–10.76)
Gertler	2008	barrett carcinoma	79 months	Europe	Southern blot	tumor tissue	2	0.53 (0.34–0.84)
Pezzolo	2015	NB tumors	43.2 months	Europe	FISH	tumor tissue	3	0.17 (0.05–0.53)
Chen	2015	glioma	24 months	Asia	qPCR	blood	2	1.39 (1.04–1.86)
Qu	2015	gastric cancer	42 months	Asia	qPCR	blood	2	2.78 (1.24–4.48)
Boscolo-Rizzo	2015	HNSCC	24 months	Europe	qPCR	tumor tissue	2	1.20 (0.67–2.14)
Disease/Progression/Treatment free survival								
Spanoudakis	2011	MPN	63 months	Europe	FISH	tumor tissue	2	0.48 (0.28–0.83)
Chen	2014	colorectal cancer	28 months	Asia	qPCR	blood	2	2.26 (1.35–3.23)
Mansouri	2013	CLL	84 months	Europe	qPCR	blood	2	1.93 (1.16–3.19)
Heaphy	2013	prostate cancer	13.2 years	America	FISH	tumor tissue	2	2.43 (1.24–4.76)
Jeon	2014	NSCLC	unclear	Asia	qPCR	tumor tissue	4	1.56 (0.76–3.20)
Lu	2011	breast cancer	86 months	Europe	qPCR	tumor tissue	2	1.20 (0.76–1.89)
Rossi	2009	CLL	32 months	Europe	Southern blot	blood	2	2.02 (1.33–3.07)
Rossi	2009	CLL	54.2 months	Europe	Southern blot	blood	2	2.14 (1.32–3.47)
Rampazzo	2012	CLL	45 months	Europe	qPCR	blood	2	2.97 (1.74–5.09)
Borssén	2011	CLL	58 months	Europe	qPCR	tumor tissue	2	0.36 (0.12–1.06)
Yan	2013	AML	12 months	Asia	Southern blot	tumor tissue	2	2.46 (1.38–4.38)
Roos	2008	CLL	52 moths	Europe	qPCR	unclear	2	1.47 (0.88–2.45)
Garcia-Aranda	2006	colorectal cancer	43.8 months	Europe	Southern blot	tumor tissue	2	0.10 (0.01–0.74)
Pezzolo	2015	NB tumors	43.2 months	Europe	FISH	tumor tissue	3	0.25 (0.10–0.63)
Augustine	2015	colorectal cancer	21 months	America	qPCR	tumor tissue	2	1.82 (1.08–3.65)
Chen	2015	glioma	24 months	Asia	qPCR	blood	2	1.41 (1.06–1.87)
Qu	2015	gastric cancer	42 months	Asia	qPCR	blood	2	2.64 (1.19–4.55)
Boscolo-Rizzo	2015	HNSCC	24 months	Europe	qPCR	tumor tissue	2	1.15 (0.59–2.25)

^a^: Mean or middle point of follow-up time.

Abbreviations: ESCC: esophageal squamous cell carcinoma, CLL: chronic lymphocytic leukemia, NSCLC: non-small-cell lung cancer, HCC: hepatocellular carcinoma, ccRCC: clear cell renal cell carcinoma, MPN: myeloproliferative neoplasms, AML: acute myelocytic leukemia, NB: neuroblastoma, HNSCC: head and neck squamous cell carcinoma, qPCR: quantitative polymerase chain reaction, FISH: fluorescence in situ hybridization-based approach.

### Results of meta-analysis

**Fig 2:**
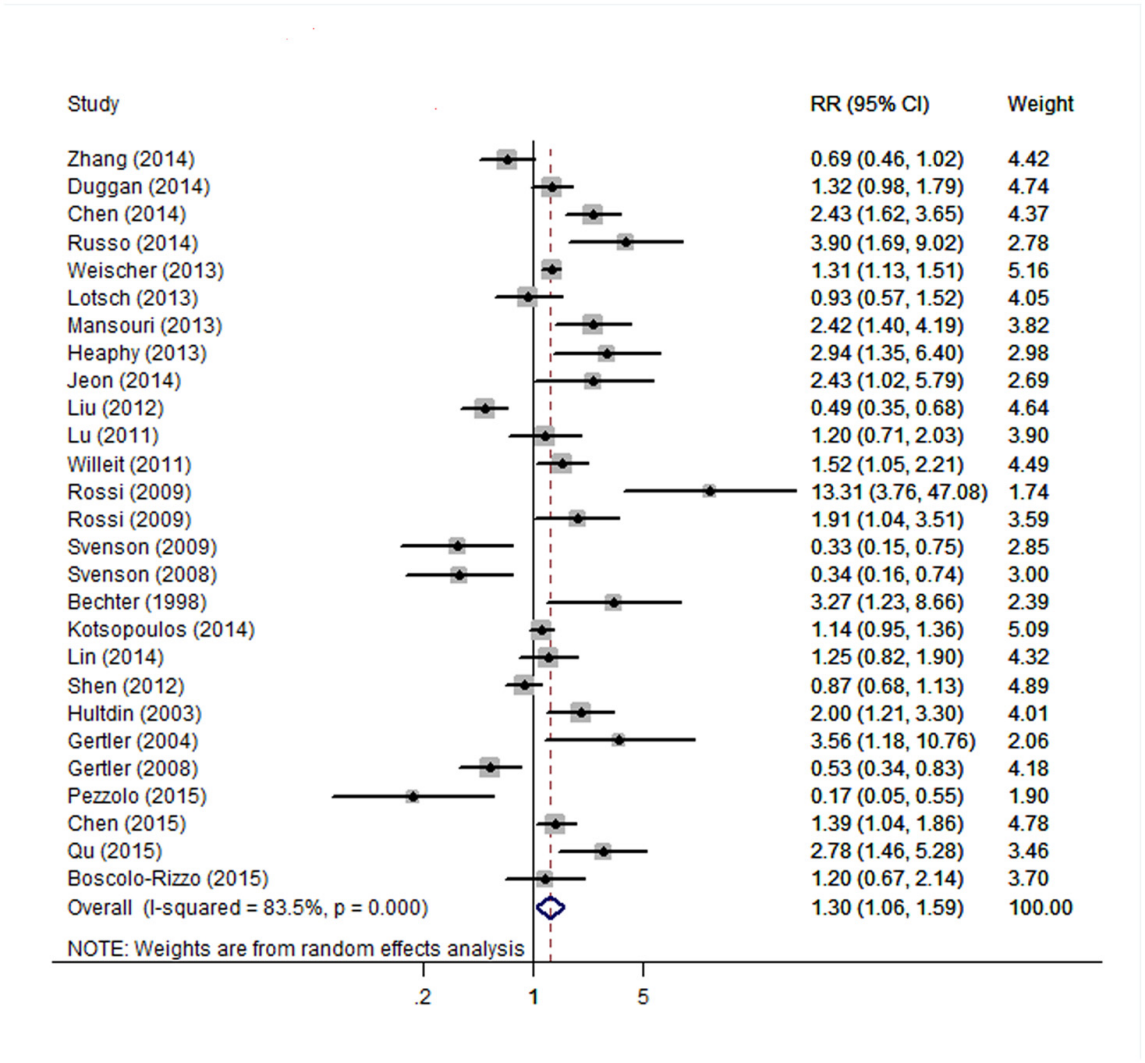
Forest plot for association between telomere length and cancer overall survival. Results are presented for random effects models.

**Fig 3:**
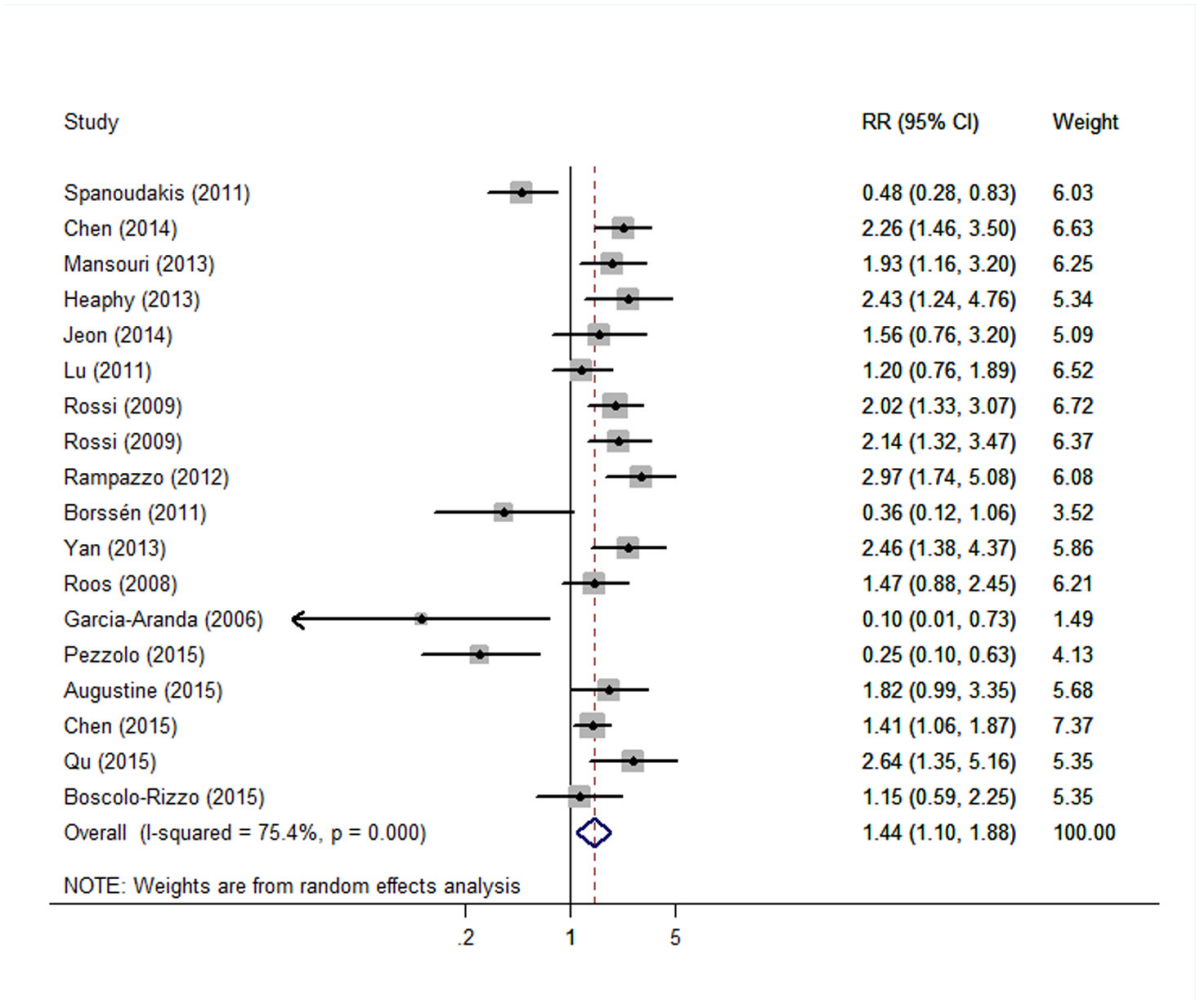
Forest plot for association between telomere length and cancer progression. Disease-free survival, treatment-free survival, progression-free survival and relapse-free survival were involved. Results are presented for random effects models.

**Fig 4:**
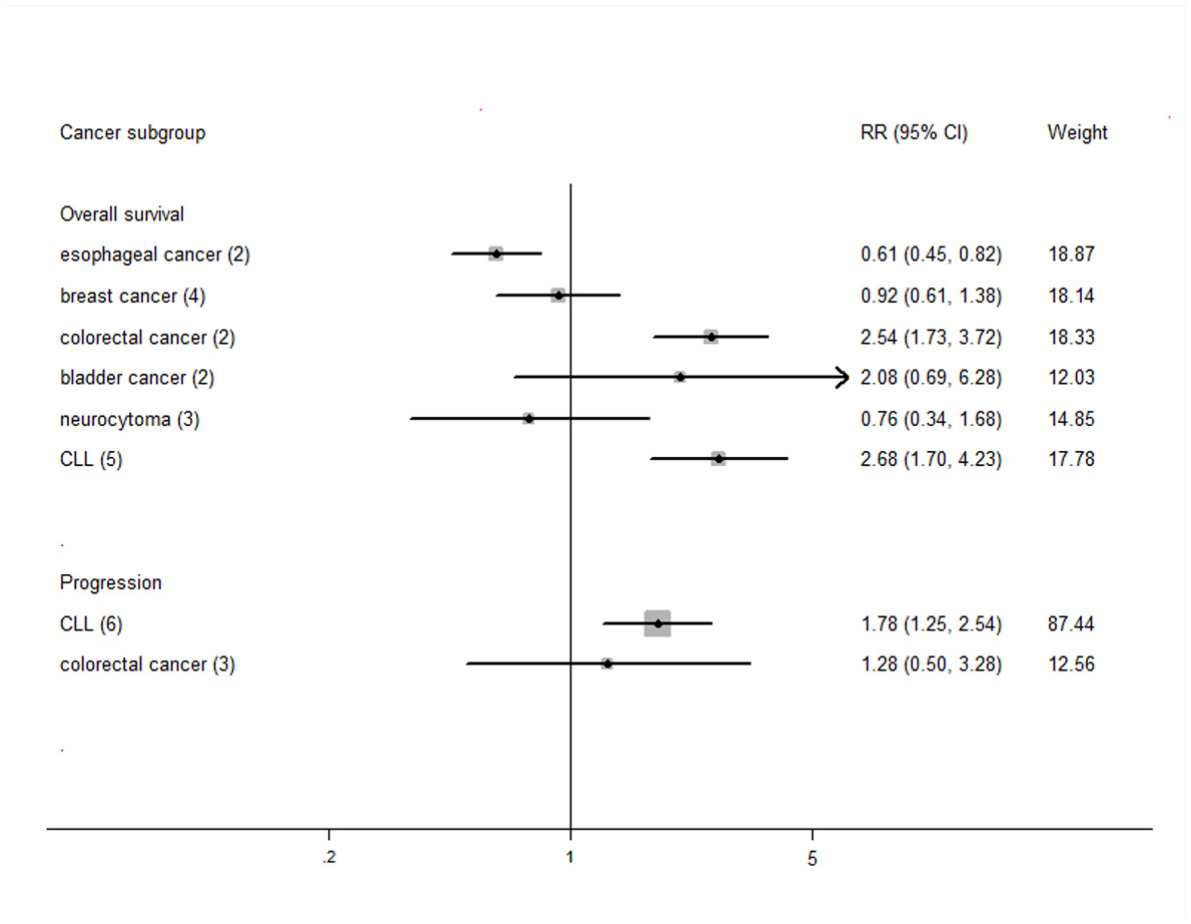
The association between telomere length and cancer prognosis in different cancers. Only cancer types included at least two studies are presented.

We observed consistent significant associations between TL and cancer overall survival and progression (Figs 2 and 3). The combined risk for cancer overall survival was 1.30 (95% CI: 1.06–1.59) for short TL compared with long TL. However, these studies displayed high heterogeneity (*I* ^*2*^ = 83.5%, *P* = 0.012). Short TL was also associated with cancer progression risk: the pooled risk estimate was 1.44 (95% CI: 1.10–1.88), with significant heterogeneity (*I* ^*2*^ = 75.4%, *P* = 0.008). However, this association varied across different cancer types (Fig 4). Short TL predicted poor overall survival and cancer progression in chronic lymphocytic leukemia, the combined RRs were 2.68 (95%CI: 1.70–4.23, *P* = 0.000, *I* ^*2*^ = 52.9%) and 1.78 (95%CI: 1.25–2.54, *P* = 0.002, *I* ^*2*^ = 61.9%), respectively. Short telomeres were also associated with increased colorectal cancer mortality (RR = 2.54, 95%CI: 1.73–3.72, *P* = 0.000, *I* ^*2*^ = 0.0%) and decreased overall survival of esophageal cancer (RR = 0.61, 95%CI: 0.45–0.82, *P* = 0.001, *I* ^*2*^ = 0.0%). We failed to find significant associations in other cancers.

### Meta-regression and subgroup analyses

Meta-regression and subgroup analyses were performed to explore the potential source of between-study heterogeneities. No subgroup presented in our study could perfectly explain the heterogeneous results for overall cancer survival (Table 2). However, when cancers were grouped into site-specific types, the results showed that short telomere was associated with increased death risk in blood cancer (RR = 2.68, 95%CI: 1.70–4.24, *P* = 0.000, *I* ^*2*^ = 52.9%), but not other cancer categories. We also found the relationship between TL and overall cancer survival was significant in studies with older subjects (RR = 1.83, 95%CI: 1.24–2.68, *P* = 0.002, *I* ^*2*^ = 70.1%), with full revisit (RR = 1.60, 95%CI: 1.11–2.30, *P* = 0.011, *I* ^*2*^ = 88.0%), with adjustment for age (RR = 1.39, 95%CI: 1.09–1.78, *P* = 0.008, *I* ^*2*^ = 76.6%), measuring TL using southern blot (RR = 2.34, 95%CI: 1.05–5.20, *P* = 0.038, *I* ^*2*^ = 86.9%), using blood samples (RR = 1.34, 95%CI: 1.05–1.70, *P* = 0.018, *I* ^*2*^ = 85.9%), dividing TL into two groups (RR = 1.30, 95%CI: 1.02–1.65, *P* = 0.035, *I* ^*2*^ = 84.6%) or four groups (RR = 1.88, 95%CI: 1.03–3.43, *P* = 0.040, *I* ^*2*^ = 91.7%) and estimating risk effects by HRs (RR = 1.43, 95%CI: 1.10–1.86, *P* = 0.008, *I* ^*2*^ = 84.9%). As for cancer progression, sample size may explain part of the heterogeneity, with *tau* ^*2*^ robustly reduced from 0.337 to 0.215 (*P* = 0.022) in a univariate meta-regression model. Additionally, *tau* ^*2*^ was reduced from 0.337 to 0.265 when stratified by risk type, suggesting that statistical method might also introduce heterogeneity (*P* = 0.050). Accordingly, only studies with larger sample size or reporting HRs remained significant after stratification, with RRs being 1.93 (95% CI: 1.58–2.36, *P* = 0.000, *I* ^*2*^ = 38.7%) and 1.69 (95% CI: 1.36–2.11, *P* = 0.000, *I* ^*2*^ = 60.1%), respectively (Table 3). In addition, short telomere predicted poor outcomes in cancer progression in studies with older participants (RR = 1.86, 95%CI: 1.37–2.54, *P* = 0.000, *I* ^*2*^ = 51.1%), with Asian or American subjects (for Asian: RR = 1.90, 95%CI: 1.44–2.51, *P* = 0.000, *I* ^*2*^ = 37.6%; for American: RR = 2.07, 95%CI: 1.32–3.26, *P* = 0.002, *I* ^*2*^ = 0.0%), with adjustment for age (RR = 1.69, 95%CI: 1.38–2.08, *P* = 0.000, *I* ^*2*^ = 20.1%), measuring TL using southern blot or qPCR (for southern blot: RR = 1.79, 95%CI: 1.04–3.10, *P* = 0.037, *I* ^*2*^ = 67.8%; for qPCR: RR = 1.64, 95%CI: 1.30–2.07, *P* = 0.000, *I* ^*2*^ = 52.4%), using blood samples (RR = 2.01, 95%CI: 1.64–2.47, *P* = 0.000, *I* ^*2*^ = 31.1%) and dichotomizing TL (RR = 1.56, 95%CI: 1.21–2.02, *P* = 0.001, *I* ^*2*^ = 72.0%).

**Table 2:** Subgroup analyses of telomere length and overall cancer survival.

Subgroup^a^	No. of studies	Risk (95% CI)	*P*	*I* ^*2*^	*P* for metareg
Age					0.323
< = 63 years	11	1.12 (0.74–1.69)	0.607	87.10%	
> 63 years	10	1.83 (1.24–2.68)	0.002	70.10%	
Unclear	6	1.17 (0.90–1.52)	0.247	83.20%	
Follow-up time					0.585
< 48 months	12	1.18 (0.77–1.81)	0.458	88.00%	
> = 48 months	10	1.60 (1.11–2.30)	0.011	81.00%	
Unclear	5	1.20 (0.91–1.58)	0.196	74.50%	
Region					0.999
Asia	6	1.35 (0.75–2.41)	0.317	91.20%	
America	5	1.20 (0.95–1.52)	0.129	63.50%	
Europe	16	1.31 (0.95–1.81)	0.098	82.70%	
Cancer category					0.177
GCs	6	1.19 (0.61–2.34)	0.611	91.80%	
Female cancers	5	1.00 (0.76–1.31)	0.990	70.20%	
Urinary cancers	3	1.17 (0.37–3.72)	0.788	88.40%	
NCs	3	0.76 (0.34–1.66)	0.484	83.90%	
Blood cancers	5	2.68 (1.70–4.24)	0.000	52.90%	
Whether adjusted for age					0.728
Yes	13	1.39 (1.09–1.78)	0.008	76.60%	
No	14	1.24 (0.88–1.75)	0.225	87.00%	
Number of participants					0.947
< 250	14	1.34 (0.89–2.02)	0.156	83.70%	
> = 250	13	1.28 (1.01–1.62)	0.043	84.60%	
Method					0.253
qPCR	19	1.19 (0.98–1.46)	0.087	82.30%	
FISH	2	0.73 (0.05–11.96)	0.827	93.60%	
Southern blot	6	2.34 (1.05–5.20)	0.038	86.90%	
DNA source					0.933
Blood	16	1.34 (1.05–1.70)	0.018	85.90%	
Tumor tissue	10	1.18 (0.76–1.83)	0.473	78.90%	
TL groups					0.365
2	22	1.30 (1.02–1.65)	0.035	84.60%	
3	2	0.55 (0.06–4.68)	0.583	60.60%	
4	3	1.88 (1.03–3.43)	0.040	91.70%	
Risk type					0.423
RR	10	1.12 (0.80–1.56)	0.525	81.00%	
HR	17	1.43 (1.10–1.86)	0.008	84.90%	
Total	27	1.30 (1.06–1.59)	0.012	83.50%	

^a^: present result if two or more studies included in a subgroup.

Abbreviations: GC: gastrointestinal cancer, NC: neurologic cancer.

**Table 3:** Subgroup analysis of telomere length and cancer progression.

Subgroup^a^	No. of studies	Risk (95% CI)	*P*	*I* ^*2*^	*P* for metareg
Age					0.609
< = 60 years	8	1.27 (0.86–1.89)	0.223	74.90%	
> 60 years	8	1.86 (1.37–2.54)	0.000	51.10%	
Unclear	2	1.05 (0.23–4.81)	0.951	94.70%	
Follow-up time					0.990
< 45 months	9	1.45 (0.99–2.13)	0.056	75.60%	
> = 45 months	8	1.40 (0.89–2.18)	0.146	80.40%	
Region					0.256
Asia	5	1.90 (1.44–2.51)	0.000	37.60%	
America	2	2.07 (1.32–3.26)	0.002	0.00%	
Europe	11	1.08 (0.70–1.66)	0.739	82.50%	
Cancer type					0.640
GCs	4	1.71 (0.90–3.25)	0.104	69.40%	
Blood cancers	8	1.51 (0.97–2.34)	0.066	80.80%	
NCs	2	0.63 (0.12–3.41)	0.591	91.90%	
Whether adjusted for age					0.254
Yes	7	1.69 (1.38–2.08)	0.000	20.10%	
No	11	1.14 (0.71–1.82)	0.589	83.60%	
Number of participants					0.022
< 170	9	0.87 (0.51–1.50)	0.612	80.40%	
> = 170	9	1.93 (1.58–2.36)	0.000	38.70%	
Method					0.210
qPCR	11	1.64 (1.30–2.07)	0.000	52.40%	
FISH	3	0.68 (0.19–2.40)	0.546	89.90%	
Southern blot	4	1.79 (1.04–3.10)	0.037	67.80%	
DNA source					0.099
Blood	7	2.01 (1.64–2.47)	0.000	31.10%	
Tumor tissue	10	0.95 (0.58–1.57)	0.854	80.10%	
TL groups					0.100
2	16	1.56 (1.21–2.02)	0.001	72.00%	
Risk type					0.050
RR	4	0.55 (0.17–1.82)	0.330	87.60%	
HR	14	1.69 (1.36–2.11)	0.000	60.10%	
Total	18	1.44 (1.10–1.88)	0.008	75.40%	

^a^: results of subgroups with two or more studies included were presented.

Abbreviations: GC: gastrointestinal cancer, NC: neurologic cancer.

### Sensitivity analysis and publication bias

**Fig 5:**
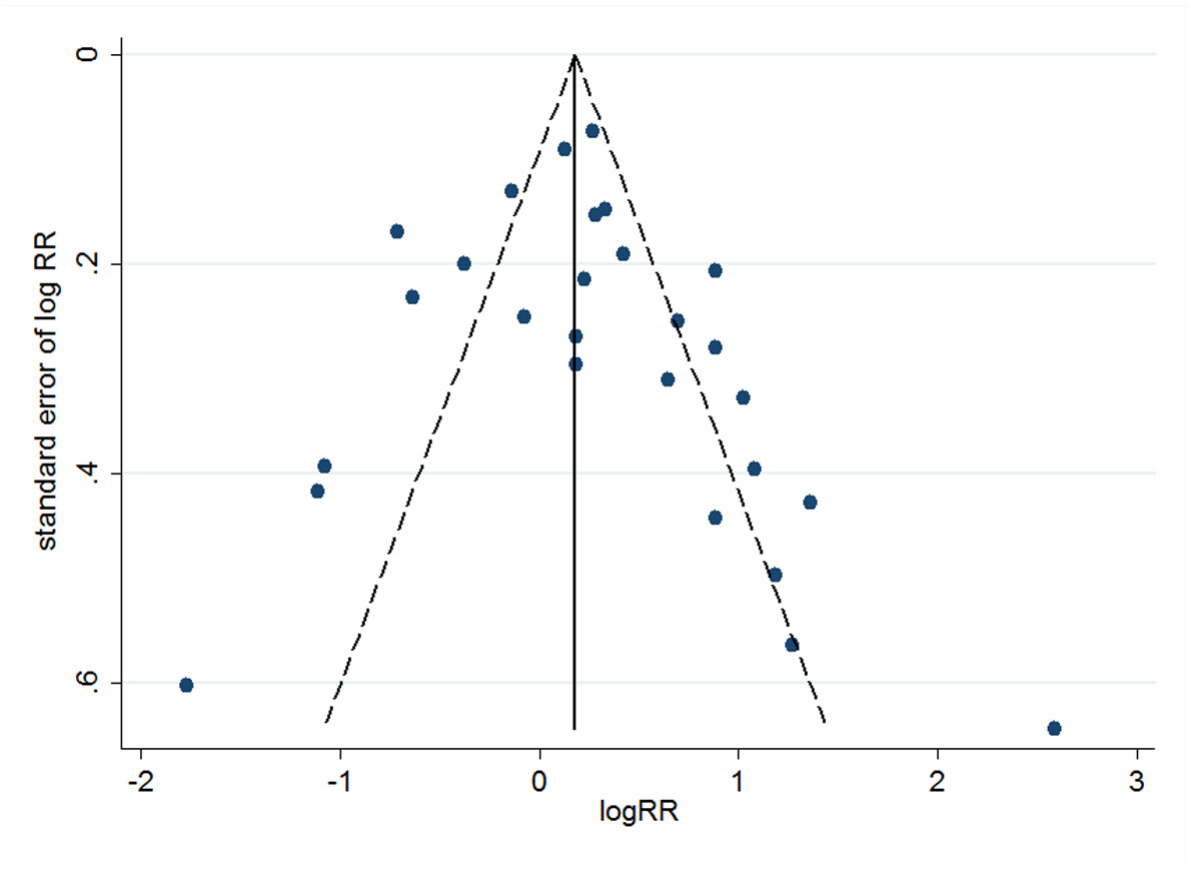
Publication bias within cancer overall survival.

Sensitivity analysis was performed subsequently. However, removing each study individually did not alter the relationships between TL and cancer outcomes materially (S2 Table). The pooled RRs for overall survival range from 1.24 (95% CI: 1.02–1.52) to 1.36 (95% CI: 1.12–1.65), and cancer progression from 1.37 (95% CI: 1.05–1.81) to 1.57 (95% CI: 1.23–2.01), while both of the relevant between-study heterogeneities remained significant. A funnel plot was drawn for twenty-seven studies which focused on overall cancer survival, and results showed no obvious asymmetry (Fig 5). On the basis of Egger’s regression test and Begg’s test, there was no evidence of publication bias among these studies (*P* = 0.431 for Egger’s test, *P* = 0.144 for Begg’s test).

## Discussion

The present meta-analyses of thirty-three independent articles, involving up to 11429 cancer patients for overall survival and 4293 cancer patients for disease progression, identified significant inverse associations between TL and cancer survival outcomes. TL was an independent predictor of prognosis in chronic lymphocytic leukemia, but not in other cancers. This association appeared to be more obvious in older populations, though it was still significant when adjusted for age. As compared with other methods (qPCR and FISH), the application of southern blot resulted in a more robust association. It also seemed that TL in blood cells was a better cancer survival predictor than TL in tumor tissue cells. In addition, large sample sizes and appropriate statistical methods guaranteed a significant association.

Telomeres play a key role in maintenance of cellular senescence and homeostasis. Over the past decades, increasing numbers of studies have observed telomere dysfunction in cancer initiation and development. Telomerase appeared to be repressed in human normal somatic cells but reactivated in cancer cells [50, 51]. Elevated telomerase mRNA expression and telomerase activity in cancer patients both predicted a poor prognosis [52, 53]. In the last few years, several single nucleotide polymorphisms (SNPs) in telomere related key genes have been identified as associated with cancer susceptibility [54–59] and cancer survival [60]. Moreover, Codd et al. showed that SNPs involved in telomere biology were associated with not only mean leukocyte TL but also with risks of several cancers and aging-associated diseases [61]. A hypothesis was that excessive telomere shortening and serious telomere uncapping could trigger DNA damage responses at chromosome ends, which were then recognized as double-strand breaks. Whether dysfunctional telomeres led to cancer or not would depend on the integrity of DNA damage responses [62]. On the other hand, critical telomere shortening was supposed to constitute a driving force for cellular transformation by causing genome instability, thus contributing to tumorigenesis [63]. As an integrated indicator of endogenous and environmental damages, TL was inversely associated with cancer susceptibility based on relevant studies and systematic reviews. Our meta-analysis suggested that TL might serve as a useful cancer prognostic biomarker and a potential therapeutic target for cancer treatment. However, the underlying mechanisms need to be clarified.

Tumorigenesis and progression are complicated processes affected by various genetic and environmental factors [64–67]. Although the present study showed a significant association between short TL and poor cancer prognosis, we still need to draw conclusions with caution based on the stratification results. Short TL may predispose to a number of cancers, but in contrast to the common pattern, longer telomeres were associated with increased risk of certain cancers, such as melanoma [68, 69] and soft tissue sarcoma [70]. Similarly, we found short telomeres predicted poor prognosis in chronic lymphocytic leukemia and colorectal cancer, but reduced death risk in esophagus cancer. Intrinsic biological heterogeneities in different cancers may be a plausible explanation. Distinct biological pathways may be influenced quite differently by TL, resulting in diverse effects on survival of different cancers. However, the number of studies included for each cancer type was far from adequate. To obtain reliable results, more relevant studies are warranted. Age is by far the predominant predictor of individual TL, explaining an estimated 17.5% of the inter-individual variation in leukocyte TL [71]. It is worth considering that TL was significantly related to cancer prognosis in studies which adjusted for age, while no significant evidence was detected in studies without age adjustment. Apparently, not only age, but also many other factors jointly contributed to cancer development and progression. We observed that if a study adjusted for age, it usually adjusted for other confounders at the same time, such as gender, smoking, stage, histology and gene expression. However, the other studies made no adjustments. Therefore, we suspected that in studies without age adjustment, other essential confounders might bias the true association. In our meta-analyses, short TL predicted terrible cancer prognosis in patients with older mean age. However, this relationship disappeared in younger patients. Recently Jeon et al. reported a similar result: shorter TL was associated with a significantly poor overall survival and disease-free survival in cancer patients more than 63 years old but not in younger patients [36]. Moreover, Cawthon et al. found telomere shortening in blood contributed to mortality in people aged 60 years or older [72]. We speculated that aged patients were vulnerable and therefore might be more sensitive to fragile telomeres than younger patients. Nevertheless, opposite and indeterminate findings also exist [25, 33]. In Asian and American subjects, significant associations were found for cancer progression, but not for overall cancer survival, while TL was neither associated with overall survival nor with cancer progression in Europeans. Owing to the high heterogeneity within studies of European subjects, it is hard to draw the negative conclusion. Besides, ethnicity might differ from person to person, even if they came from the same country. However, we could not investigate this relationship because there was insufficient information.

Other factors including study design, experimental approach and analysis strategy can also influence the detection of the relationships between TL and cancers. As compared to qPCR, southern blot conferred a modestly stronger effect for both overall cancer survival and progression. Unlike direct measurement such as by southern blot, qPCR evaluate TL using a ratio of telomere/single copy gene (T/S) [73]. This indirect method can’t generate absolute values for TL, and thus may introduce measurement error and bias real effects. TL in blood cells showed a stronger relationship with age compared with TL in other tissues, whether in healthy populations or in cancer patients [72, 74]. Our results were in agreement with this and suggested that leucocyte TL might be a preferential indicator for age-related disease and disease survival. This was exciting because blood is a convenient tissue to collect and yields high-quality DNA which is more suitable for TL assays than that from other tumor or non-tumor tissues. In the great majority of the included studies, telomeres were divided into two groups: a long telomere group and a short telomere group. Although the classification criteria might be different (e.g. median of TL, cut-point from ROC curve or empirical value), our results showed that dichotomizing TL was sufficient to find a significant association. However, association was non-significant in studies which divided telomeres into three groups. Since only two studies were in this subgroup, we think more studies are needed. In addition, studies with larger sample sizes and estimating risk effects using HRs explained part of the heterogeneity among studies for cancer progression and shared consistent significant associations for overall cancer survival and progression, indicating that well designed large cohort studies are necessary to discover the true associations.

As far as we are aware, this is the first meta-analysis discussing the association between TL and cancer survival. The included articles were all prospective studies, and the records of TL and disease outcomes were considered credible. Our results seemed stable and reliable after performing sensitivity analyses and testing the publication bias. Despite its strengths, some limitations should be acknowledged. Firstly, we failed to find definitive sources of heterogeneity in the studies for cancer overall survival. Discords existed in the results of several subgroups after stratification, for example, TL was significantly associated with cancer overall survival in studies with older mean age, but not in younger patients. However, these discrepancies cannot be the cause of the heterogeneity as there was high heterogeneity among the studies in each subgroup. Although we have identified a partial source of heterogeneity for cancer progression, we could not investigate further due to limited information. Secondly, TLs were measured at a single time point in our included studies. A series of longitudinal TL records might contribute to characterization of the dynamic variation of this association. Lastly, cancer survival was determined by many factors, including cancer stage, pathological pattern, and critically, disease therapy. Only discussing age in our stratification analyses could be considered insufficient. More large scale studies are required to further explore the association and uncover the underlying biological mechanism.

In conclusion, our meta-analyses provided evidence for an inverse association between TL and risk of cancer survival, particularly for chronic lymphocytic leukemia. The association suggested that leucocyte TL might be a predictive biomarker for cancer prognosis in old people. However, our findings need to be updated and confirmed in the future, and additional studies are required to further characterize the nature of this association under different circumstances.

## Supporting Information

S1 File PRISMA checklist: Preferred Reporting Items for Systematic Reviews and Meta-Analyses. (DOC)

S1 Table Adjusted confounders for each study. (DOCX)

S2 Table Results of sensitivity analysis. (DOCX)

## References

[1] Blackburn EH. Structure and function of telomeres. Nature 1991; 350: 569–573. 1708110 10.1038/350569a0

[2] Blackburn EH. Switching and signaling at the telomere. Cell 2001; 106: 661–673. 11572773 10.1016/s0092-8674(01)00492-5

[3] Huffman KE, Levene SD, Tesmer VM, Shay JW, Wright WE. Telomere shortening is proportional to the size of the G-rich telomeric 3'-overhang. J Biol Chem. 2000; 275: 19719–19722. 10787419 10.1074/jbc.M002843200

[4] Harley CB. Telomere loss: mitotic clock or genetic time bomb? Mutat Res. 1991; 256: 271–282. 1722017 10.1016/0921-8734(91)90018-7

[5] Martinez P, Blasco MA. Telomeric and extra-telomeric roles for telomerase and the telomere-binding proteins. Nat Rev Cancer 2011; 11: 161–176. 10.1038/nrc3025 21346783

[6] Sanders JL, Newman AB. Telomere length in epidemiology: a biomarker of aging, age-related disease, both, or neither? Epidemiol Rev. 2013; 35: 112–131. 10.1093/epirev/mxs008 23302541 PMC4707879

[7] Artandi SE, Chang S, Lee SL, Alson S, Gottlieb GJ, Chin L, et al Telomere dysfunction promotes non-reciprocal translocations and epithelial cancers in mice. Nature 2000; 406: 641–645. 10949306 10.1038/35020592

[8] Blasco MA, Lee HW, Hande MP, Samper E, Lansdorp PM, DePinho RA, et al Telomere shortening and tumor formation by mouse cells lacking telomerase RNA. Cell 1997; 91: 25–34. 9335332 10.1016/s0092-8674(01)80006-4

[9] Engelhardt M, Drullinsky P, Guillem J, Moore MA. Telomerase and telomere length in the development and progression of premalignant lesions to colorectal cancer. Clin Cancer Res. 1997; 3: 1931–1941. 9815582

[10] Hastie ND, Dempster M, Dunlop MG, Thompson AM, Green DK, Allshire RC. Telomere reduction in human colorectal carcinoma and with ageing. Nature 1990; 346: 866–868. 2392154 10.1038/346866a0

[11] Rampazzo E, Bertorelle R, Serra L, Terrin L, Candiotto C, Pucciarelli S, et al Relationship between telomere shortening, genetic instability, and site of tumour origin in colorectal cancers. Br J Cancer 2010; 102: 1300–1305. 10.1038/sj.bjc.6605644 20386541 PMC2856015

[12] D'Mello MJ, Ross SA, Briel M, Anand SS, Gerstein H, Paré G. Association between shortened leukocyte telomere length and cardiometabolic outcomes: systematic review and meta-analysis. Circ Cardiovasc Genet. 2015; 8: 82–90. 10.1161/CIRCGENETICS.113.000485 25406241

[13] Haycock PC, Heydon EE, Kaptoge S, Butterworth AS, Thompson A, Willeit P. Leucocyte telomere length and risk of cardiovascular disease: systematic review and meta-analysis. BMJ 2014; 349: g4227–g4227. 10.1136/bmj.g4227 25006006 PMC4086028

[14] Wentzensen IM, Mirabello L, Pfeiffer RM, Savage SA. The association of telomere length and cancer: a meta-analysis. Cancer Epidemiol Biomarkers Prev. 2011; 20: 1238–1250. 10.1158/1055-9965.EPI-11-0005 21467229 PMC3111877

[15] Ma H, Zhou Z, Wei S, Liu Z, Pooley KA, Dunning AM, et al Shortened telomere length is associated with increased risk of cancer: a meta-analysis. PLoS One 2011; 6: e20466 10.1371/journal.pone.0020466 21695195 PMC3112149

[16] Chen Y, Qu F, He X, Bao G, Liu X, Wan S, et al Short leukocyte telomere length predicts poor prognosis and indicates altered immune functions in colorectal cancer patients. Ann Oncol. 2014; 25: 869–876. 10.1093/annonc/mdu016 24608194

[17] Garcia-Aranda C, de Juan C, Diaz-Lopez A, Sanchez-Pernaute A, Torres A-J, Diaz-Rubio E, et al Correlations of telomere length, telomerase activity, and telomeric-repeat binding factor 1 expression in colorectal carcinoma. Cancer 2006; 106: 541–551. 16388518 10.1002/cncr.21625

[18] Svenson U, Nordfjall K, Stegmayr B, Manjer J, Nilsson P, Tavelin B, et al Breast cancer survival is associated with telomere length in peripheral blood cells. Cancer Res. 2008; 68: 3618–3623. 10.1158/0008-5472.CAN-07-6497 18483243

[19] Duggan C, Risques R, Alfano C, Prunkard D, Imayama I, Holte S, et al Change in peripheral blood leukocyte telomere length and mortality in breast cancer survivors. J Natl Cancer Inst. 2014; 106: dju035 10.1093/jnci/dju035 24627273 PMC3982887

[20] Lu L, Zhang C, Zhu G, Irwin M, Risch H, Menato G, et al Telomerase expression and telomere length in breast cancer and their associations with adjuvant treatment and disease outcome. Breast Cancer Res. 2011; 13: R56 10.1186/bcr2893 21645396 PMC3218945

[21] Shen J, Gammon MD, Terry MB, Bradshaw PT, Wang Q, Teitelbaum SL, et al Genetic polymorphisms in telomere pathway genes, telomere length, and breast cancer survival. Breast Cancer Res Treat. 2012; 134: 393–400. 10.1007/s10549-012-2058-9 22527105 PMC3579614

[22] Wells G, Shea B, O’Connell D, Peterson J, Welch V, Losos M, et al The Newcastle-Ottawa Scale (NOS) for assessing the quality of nonrandomised studies in metaanalyses. Ottawa Health Research Institute Available: http://www.ohri.ca/programs/clinical_epidemiology/oxford.asp.

[23] Zhang D-H, Chen J-Y, Hong C-Q, Yi D-Q, Wang F, Cui W. High-risk human papillomavirus infection associated with telomere elongation in patients with esophageal squamous cell carcinoma with poor prognosis. Cancer 2014; 120: 2673–2683. 10.1002/cncr.28797 24840723

[24] Yan S, Han B, Wu Y, Zhou D, Zhao Y. Telomerase gene mutation screening and telomere overhang detection in Chinese patients with acute myeloid leukemia. Leuk Lymphoma 2013; 54: 1437–1441. 10.3109/10428194.2012.729834 23157242

[25] Weischer M, Nordestgaard BG, Cawthon RM, Freiberg JJ, Tybjaerg-Hansen A, Bojesen SE. Short telomere length, cancer survival, and cancer risk in 47102 individuals. J Natl Cancer Inst. 2013; 105: 459–468. 10.1093/jnci/djt016 23468462

[26] Svenson U, Ljungberg B, Roos G. Telomere length in peripheral blood predicts survival in clear cell renal cell carcinoma. Cancer Res. 2009; 69: 2896–2901. 10.1158/0008-5472.CAN-08-3513 19318563

[27] Spanoudakis E, Bazdiara I, Pantelidou D, Kotsianidis I, Papadopoulos V, Margaritis D, et al Dynamics of telomere's length and telomerase activity in Philadelphia chromosome negative myeloproliferative neoplasms. Leuk Res. 2011; 35: 459–464. 10.1016/j.leukres.2010.07.042 20828816

[28] Russo A, Modica F, Guarrera S, Fiorito G, Pardini B, Viberti C, et al Shorter leukocyte telomere length is independently associated with poor survival in patients with bladder cancer. Cancer Epidemiol Biomarkers Prev. 2014; 23: 2439–2446. 10.1158/1055-9965.EPI-14-0228 25234236

[29] Rossi D, Lobetti Bodoni C, Genuardi E, Monitillo L, Drandi D, Cerri M, et al Telomere length is an independent predictor of survival, treatment requirement and Richter's syndrome transformation in chronic lymphocytic leukemia. Leukemia 2009; 23: 1062–1072. 10.1038/leu.2008.399 19340005

[30] Rampazzo E, Bonaldi L, Trentin L, Visco C, Keppel S, Giunco S, et al Telomere length and telomerase levels delineate subgroups of B-cell chronic lymphocytic leukemia with different biological characteristics and clinical outcomes. Haematologica. 2011; 97: 56–63. 10.3324/haematol.2011.049874 21933855 PMC3248931

[31] Mansouri L, Grabowski P, Degerman S, Svenson U, Gunnarsson R, Cahill N, et al Short telomere length is associated with NOTCH1/SF3B1/TP53 aberrations and poor outcome in newly diagnosed chronic lymphocytic leukemia patients. Am J Hematol. 2013; 88: 647–651. 10.1002/ajh.23466 23620080

[32] Lotsch D, Ghanim B, Laaber M, Wurm G, Weis S, Lenz S, et al Prognostic significance of telomerase-associated parameters in glioblastoma: effect of patient age. Neuro Oncol. 2013; 15: 423–432. 10.1093/neuonc/nos329 23393205 PMC3607268

[33] Liu HQ, An JZ, Liu J, Yang YF, Zhang HX, Zhao BY, et al Leukocyte telomere length predicts overall survival in hepatocellular carcinoma treated with transarterial chemoembolization. Carcinogenesis 2012; 33: 1040–1045. 10.1093/carcin/bgs098 22318909 PMC6276896

[34] Lin J, Blalock JA, Chen M, Ye Y, Gu J, Cohen L, et al Depressive symptoms and short telomere length are associated with increased mortality in bladder cancer patients. Cancer Epidemiol Biomarkers Prev. 2015; 24: 336–343. 10.1158/1055-9965.EPI-14-0992 25416716 PMC4332382

[35] Kotsopoulos J, Prescott J, De Vivo I, Fan I, Mclaughlin J, Rosen B, et al Telomere length and mortality following a diagnosis of ovarian cancer. Cancer Epidemiol Biomarkers Prev. 2014; 23: 2603–2606. 10.1158/1055-9965.EPI-14-0885 25159293 PMC4221534

[36] Jeon H-S, Choi YY, Choi JE, Lee WK, Lee E, Yoo SS, et al Telomere length of tumor tissues and survival in patients with early stage non-small cell lung cancer. Mol Carcinog. 2014; 53: 272–279. 10.1002/mc.21972 23065897

[37] Hultdin M, Rosenquist R, Thunberg U, Tobin G, Norrback KF, Johnson A, et al Association between telomere length and VH gene mutation status in chronic lymphocytic leukaemia: clinical and biological implications. Br J Cancer 2003; 88: 593–598. 12592375 10.1038/sj.bjc.6600763 PMC2377180

[38] Heaphy CM, Yoon GS, Peskoe SB, Joshu CE, Lee TK, Giovannucci E, et al Prostate cancer cell telomere length variability and stromal cell telomere length as prognostic markers for metastasis and death. Cancer Discov. 2013; 3: 1130–1141. 10.1158/2159-8290.CD-13-0135 23779129 PMC3797255

[39] Gertler R, Doll D, Maak M, Feith M, Rosenberg R. Telomere length and telomerase subunits as diagnostic and prognostic biomarkers in Barrett carcinoma. Cancer 2008; 112: 2173–2180. 10.1002/cncr.23419 18348304

[40] Gertler R. Telomere length and human telomerase reverse transcriptase expression as markers for progression and prognosis of colorectal carcinoma. J Clin Oncol. 2004; 22: 1807–1814. 15143073 10.1200/JCO.2004.09.160

[41] Borssén M, Cullman I, Norén-Nyström U, Sundström C, Porwit A, Forestier E, et al hTERT promoter methylation and telomere length in childhood acute lymphoblastic leukemia-associations with immunophenotype and cytogenetic subgroup. Exp Hematol. 2011; 39: 1144–1151. 10.1016/j.exphem.2011.08.014 21914494

[42] Willeit P, Willeit J, Kloss-Brandstatter A, Kronenberg F, Kiechl S. Fifteen-year follow-up of association between telomere length and incident cancer and cancer mortality. JAMA 2011; 306: 42–44. 10.1001/jama.2011.901 21730239

[43] Roos G, Kröber A, Grabowski P, Kienle D, Bühler A, Döhner H, et al Short telomeres are associated with genetic complexity, high-risk genomic aberrations, and short survival in chronic lymphocytic leukemia. Blood 2008; 111: 2246–2252. 18045969 10.1182/blood-2007-05-092759

[44] Bechter OE, Eisterer W, Pall G, Hilbe W, Kühr T, Thaler J, et al Telomere length and telomerase activity predict survival in patients with B cell chronic lymphocytic leukemia. Cancer Res. 1998; 58: 4918–4922. 9810000

[45] Augustine TA, Baig M, Sood A, Budagov T, Atzmon G, Mariadason JM, et al Telomere length is a novel predictive biomarker of sensitivity to anti-EGFR therapy in metastatic colorectal cancer. Br J Cancer 2015; 112: 313–318. 10.1038/bjc.2014.561 25412235 PMC4453445

[46] Pezzolo A, Pistorio A, Gambini C, Haupt R, Ferraro M, Erminio G, et al Intratumoral diversity of telomere length in individual neuroblastoma tumors. Oncotarget 2015; 6: 7493–7503. 25595889 10.18632/oncotarget.2115 PMC4480695

[47] Chen Y, Wu Y, Huang X, Qu P, Li G, Jin T, et al Leukocyte telomere length: a novel biomarker to predict the prognosis of glioma patients. J Cancer Res Clin Oncol. 2015; In press. 10.1007/s00432-015-1938-x PMC11823758 25702101

[48] Qu F, Li R, He X, Li Q, Xie S, Gong L, et al Short telomere length in peripheral blood leukocyte predicts poor prognosis and indicates an immunosuppressive phenotype in gastric cancer patients. Mol Oncol. 2015; 9: 727–739. 10.1016/j.molonc.2014.11.008 25515040 PMC5528698

[49] Boscolo-Rizzo P, Rampazzo E, Perissinotto E, Piano MA, Giunco S, Baboci L, et al Telomere shortening in mucosa surrounding the tumor: biosensor of field cancerization and prognostic marker of mucosal failure in head and neck squamous cell carcinoma. Oral Oncol. 2015; 51: 500–507. 10.1016/j.oraloncology.2015.02.100 25771075

[50] Kim NW, Piatyszek MA, Prowse KR, Harley CB, West MD, Ho PL, et al Specific association of human telomerase activity with immortal cells and cancer. Science 1994; 266: 2011–2015. 7605428 10.1126/science.7605428

[51] Tahara H, Kuniyasu H, Yokozaki H, Yasui W, Shay JW, Ide T, et al Telomerase activity in preneoplastic and neoplastic gastric and colorectal lesions. Clin Cancer Res. 1995; 1: 1245–1251. 9815918

[52] Bertorelle R, Briarava M, Rampazzo E, Biasini L, Agostini M, Maretto I, et al Telomerase is an independent prognostic marker of overall survival in patients with colorectal cancer. Br J Cancer 2013; 108: 278–284. 10.1038/bjc.2012.602 23322193 PMC3566802

[53] Gertler R, Rosenberg R, Stricker D, Friederichs J, Hoos A, Werner M, et al Telomere length and human telomerase reverse transcriptase expression as markers for progression and prognosis of colorectal carcinoma. J Clin Oncol. 2004; 22: 1807–1814. 15143073 10.1200/JCO.2004.09.160

[54] Haiman CA, Chen GK, Vachon CM, Canzian F, Dunning A, Millikan RC, et al A common variant at the TERT-CLPTM1L locus is associated with estrogen receptor-negative breast cancer. Nat Genet. 2011; 43: 1210–1214. 10.1038/ng.985 22037553 PMC3279120

[55] Kinnersley B, Migliorini G, Broderick P, Whiffin N, Dobbins SE, Casey G, et al The TERT variant rs2736100 is associated with colorectal cancer risk. Br J Cancer 2012; 107: 1001–1008. 10.1038/bjc.2012.329 22878375 PMC3464867

[56] McKay JD, Hung RJ, Gaborieau V, Boffetta P, Chabrier A, Byrnes G, et al Lung cancer susceptibility locus at 5p15.33. Nat Genet. 2008; 40: 1404–1406. 10.1038/ng.254 18978790 PMC2748187

[57] Petersen GM, Amundadottir L, Fuchs CS, Kraft P, Stolzenberg-Solomon RZ, Jacobs KB, et al A genome-wide association study identifies pancreatic cancer susceptibility loci on chromosomes 13q22.1, 1q32.1 and 5p15.33. Nat Genet. 2010; 42: 224–228. 10.1038/ng.522 20101243 PMC2853179

[58] Shete S, Hosking FJ, Robertson LB, Dobbins SE, Sanson M, Malmer B, et al Genome-wide association study identifies five susceptibility loci for glioma. Nat Genet. 2009; 41: 899–904. 10.1038/ng.407 19578367 PMC4501476

[59] Zhong R, Liu L, Zou L, Zhu Y, Chen W, Zhu B, et al Genetic variations in TERT-CLPTM1L locus are associated with risk of lung cancer in Chinese population. Mol Carcinog. 2013; 52 Suppl 1: E118–126. 10.1002/mc.22043 23908149

[60] Rachakonda PS, Hosen I, de Verdier PJ, Fallah M, Heidenreich B, Ryk C, et al TERT promoter mutations in bladder cancer affect patient survival and disease recurrence through modification by a common polymorphism. Proc Natl Acad Sci U S A 2013; 110: 17426–17431. 10.1073/pnas.1310522110 24101484 PMC3808633

[61] Codd V, Nelson CP, Albrecht E, Mangino M, Deelen J, Buxton JL, et al Identification of seven loci affecting mean telomere length and their association with disease. Nat Genet. 2013; 45: 422–427, e421–422. 10.1038/ng.2528 23535734 PMC4006270

[62] de Lange T. How telomeres solve the end-protection problem. Science 2009; 326: 948–952. 10.1126/science.1170633 19965504 PMC2819049

[63] Günes C, Rudolph KL. The role of telomeres in stem cells and cancer. Cell 2013; 152: 390–393. 10.1016/j.cell.2013.01.010 23374336

[64] Nelson HD, Zakher B, Cantor A, Fu R, Griffin J, O'Meara ES, et al Risk factors for breast cancer for women aged 40 to 49 years: a systematic review and meta-analysis. Ann Intern Med. 2012; 156: 635–648. 10.7326/0003-4819-156-9-201205010-00006 22547473 PMC3561467

[65] Zhu B, Tian J, Zhong R, Tian Y, Chen W, Qian J, et al Genetic variants in the SWI/SNF complex and smoking collaborate to modify the risk of pancreatic cancer in a Chinese population. Mol Carcinog. 2014; In press. 10.1002/mc.22140 24585446

[66] Li J, Zou L, Chen W, Zhu B, Shen N, Ke J, et al Dietary mushroom intake may reduce the risk of breast cancer: evidence from a meta-analysis of observational studies. PLoS One 2014; 9: e93437 10.1371/journal.pone.0093437 24691133 PMC3972098

[67] Zhu B, Zou L, Qi L, Zhong R, Miao X. Allium vegetables and garlic supplements do not reduce risk of colorectal cancer, based on meta-analysis of prospective studies. Clin Gastroenterol Hepatol. 2014; 12: 1991–2001, e1–4. 10.1016/j.cgh.2014.03.019 24681077

[68] Burke LS, Hyland PL, Pfeiffer RM, Prescott J, Wheeler W, Mirabello L, et al Telomere length and the risk of cutaneous malignant melanoma in melanoma-prone families with and without CDKN2A mutations. PLoS One 2013; 8: e71121 10.1371/journal.pone.0071121 23990928 PMC3747185

[69] Nan H, Du M, De Vivo I, Manson JE, Liu S, McTiernan A, et al Shorter telomeres associate with a reduced risk of melanoma development. Cancer Res. 2011; 71: 6758–6763. 10.1158/0008-5472.CAN-11-1988 22028319 PMC3206204

[70] Xie H, Wu X, Wang S, Chang D, Pollock RE, Lev D, et al Long telomeres in peripheral blood leukocytes are associated with an increased risk of soft tissue sarcoma. Cancer 2013; 119: 1885–1891. 10.1002/cncr.27984 23408253 PMC3807211

[71] Daniali L, Benetos A, Susser E, Kark JD, Labat C, Kimura M, et al Telomeres shorten at equivalent rates in somatic tissues of adults. Nat Commun. 2013; 4: 1597 10.1038/ncomms2602 23511462 PMC3615479

[72] Cawthon RM, Smith KR, O'Brien E, Sivatchenko A, Kerber RA. Association between telomere length in blood and mortality in people aged 60 years or older. Lancet 2003; 361: 393–395. 12573379 10.1016/S0140-6736(03)12384-7

[73] Cawthon RM. Telomere measurement by quantitative PCR. Nucleic Acids Res. 2003; 30: e47. 10.1093/nar/30.10.e47 PMC115301 12000852

[74] Valls-Bautista C, Piñol-Felis C, Reñé-Espinet JM, Buenestado-García J, Viñas-Salas J. In colon cancer, normal colon tissue and blood cells have altered telomere lengths. J Surg Oncol. 2015; 111: 899–904. 10.1002/jso.23894 25873347

