# Supplementary material for: The Association between Telomere Length and Cancer Prognosis: Evidence from a Meta-Analysis
Source: PLoS One. 2015 Jul 15;10(7):e0133174. doi: 10.1371/journal.pone.0133174 (PMC4503690; doi:10.1371/journal.pone.0133174)
Supplement: S1 Table — (DOCX) [file pone.0133174.s002.docx]

**S1 Table. Adjusted confounders for each study**

| First author | Year | | Cancer | Adjusted confounders |
| --- | --- | --- | --- | --- |
| Overall survival | |  |  |  |
| Zhang | 2014 | | ESCC | NA |
| Duggan | 2014 | | breast cancer | NA |
| Chen | 2014 | | colorectal cancer | NA |
| Russo | 2014 | | bladder cancer | age, TG, BCG, radical cystectomy, radiotherapy, and chemotherapy |
| Weischer | 2013 | | multiple cancers | age at study entry, sex, year of birth, current smoking, cumulative smoking,BMI, heavy alcohol intake |
| Lötsch | 2013 | | glioblastoma | age, sex, KPS, treatment, and IDH1 mutation status |
| Mansouri | 2013 | | CLL | age, binet stage, gender, CD38 expression, Trisomy 12, del(11q), TP53 alteration/NOTCH1 mut/SF3B1 mut |
| Heaphy | 2013 | | prostate cancer | age and year of diagnosis, prostatectomy Gleason sum, pathologic TNM stage, and serum PSA concentration at diagnosis |
| Jeon | 2014 | | NSCLC | age, gender, somking status, histology, pathologic stage |
| Liu | 2012 | | HCC | tumor size, PVT, Child–Pugh score, and AFP |
| Lu | 2011 | | breast cancer | age at surgery, disease stage, tumor grade, histological type, estrogen- and progesterone-receptor status |
| Willeit | 2011 | | multiple cancers | NA |
| Rossi | 2009 | | CLL | IGHV gene homology and Binet stage |
| Rossi | 2009 | | CLL | IGHV gene homology, unfavorable FISH karyotype, age, Binet stage and b-2-microglobulin levels. |
| Svenson | 2009 | | ccRCC | age, TNM stage |
| Svenson | 2008 | | breast cancer | age |
| Bechter | 1998 | | CLL | NA |
| Kotsopoulos | 2014 | | ovarian cancer | age, batch, stage, age at diagnosis, BRCA mutation status, histologic subtype, body mass, smoking history, and chemotherapy. |
| Lin | 2014 | | bladder cancer | NA |
| Shen | 2012 | | breast cancer | NA |
| Hultdin | 2003 | | CLL | NA |
| Gertler | 2004 | | colorectal cancer | NA |
| Gertler | 2008 | | barrett carcinoma | NA |
| Pezzolo | 2015 | | NB tumors | NA |
| Chen | 2015 | | glioma | age, gender, grade, karnofsky performance score, surgery, and adjuvant therapy |
| Qu | 2015 | | gastric cancer | age, sex, tumor site, TNM stage, differentation, Lauren classification, and chemotherapy |
| Boscolo-Rizzo | 2015 | | HNSCC | NA |
| Disease/Progression/Treatment free survival | | | |  |
| Spanoudakis | 2011 | | MPN | NA |
| Chen | 2014 | | colorectal cancer | NA |
| Mansouri | 2013 | | CLL | age, binet stage, gender, CD38 expression, Trisomy 12, del(11q), TP53 alteration/NOTCH1 mut/SF3B1 mut |
| Heaphy | 2013 | | prostate cancer | age and year of diagnosis, prostatectomy Gleason sum, pathologic TNM stage, and serum PSA concentration at diagnosis |
| Jeon | 2014 | | NSCLC | age, gender, somking status, histology, pathologic stage |
| Lu | 2011 | | breast cancer | age at surgery, disease stage, tumor grade, histological type, estrogen- and progesterone-receptor status |
| Rossi | 2009 | | CLL | IGHV gene homology, age, sex and Binet stage |
| Rossi | 2009 | | CLL | IGHV gene homology, unfavorable FISH karyotype, CD38 expression, ZAP70 expression, Binet stage, peripheral blood lymphocytes and b-2-microglobulin levels. |
| Rampazzo | 2012 | | CLL | NA |
| Borssén | 2011 | | CLL | NA |
| Yan | 2013 | | AML | NA |
| Roos | 2008 | | CLL | ZAP-70, CD38, IGHV mutation status, High-risk genomic aberrations |
| Garcia-Aranda | 2006 | | colorectal cancer | NA |
| Pezzolo | 2015 | | NB tumors | stage, h-TERT expression |
| Augustine | 2015 | | colorectal cancer | NA |
| Chen | 2015 | | glioma | age, gender, grade, karnofsky performance score, surgery, and adjuvant therapy |
| Qu | 2015 | | gastric cancer | age, sex, tumor site, TNM stage, differentation, Lauren classification, and chemotherapy |
| Boscolo-Rizzo | 2015 | | HNSCC | NA |

Abbreviations: ESCC: esophageal squamous cell carcinoma, CLL: chronic lymphocytic leukemia, NSCLC: non-small-cell lung cancer, HCC: hepatocellular carcinoma, ccRCC: clear cell renal cell carcinoma, MPN: myeloproliferative neoplasms, AML: acute myelocytic leukemia, NB: neuroblastoma, HNSCC: head and neck squamous cell carcinoma
