# Supplementary material for: The Association between Telomere Length and Cancer Prognosis: Evidence from a Meta-Analysis
Source: PLoS One. 2015 Jul 15;10(7):e0133174. doi: 10.1371/journal.pone.0133174 (PMC4503690; doi:10.1371/journal.pone.0133174)
Supplement: S2 Table — (DOCX) [file pone.0133174.s003.docx]

**S2 Table. Results of sensitivity analysis**

| Study ommited | Cancer | RR | 95% CI | *P* | I ^2^ | *P*_hetero_ |
| --- | --- | --- | --- | --- | --- | --- |
| Overall survival |  |  |  |  |  |  |
| Zhang 2014 | ESCC | 1.34 | 1.09-1.64 | 0.006 | 83.3% | 0.000 |
| Duggan 2014 | breast cancer | 1.30 | 1.05-1.61 | 0.016 | 84.1% | 0.000 |
| Chen 2014 | colorectal cancer | 1.26 | 1.03-1.54 | 0.027 | 82.9% | 0.000 |
| Russo 2014 | bladder cancer | 1.26 | 1.03-1.54 | 0.027 | 83.3% | 0.000 |
| Weischer 2013 | multiple cancers | 1.31 | 1.04-1.65 | 0.022 | 84.0% | 0.000 |
| Lötsch 2013 | glioblastoma | 1.32 | 1.07-1.63 | 0.010 | 84.0% | 0.000 |
| Mansouri 2013 | CLL | 1.27 | 1.03-1.55 | 0.025 | 83.5% | 0.000 |
| Heaphy 2013 | prostate cancer | 1.27 | 1.03-1.55 | 0.024 | 83.6% | 0.000 |
| Jeon 2014 | NSCLC | 1.28 | 1.04-1.57 | 0.020 | 83.9% | 0.000 |
| Liu 2012 | HCC | 1.36 | 1.12-1.65 | 0.002 | 80.5% | 0.000 |
| Lu 2011 | breast cancer | 1.30 | 1.06-1.61 | 0.013 | 84.2% | 0.000 |
| Willeit 2011 | multiple cancers | 1.29 | 1.05-1.59 | 0.018 | 84.0% | 0.000 |
| Rossi 2009-1 | CLL | 1.24 | 1.02-1.52 | 0.030 | 82.6% | 0.000 |
| Rossi 2009-2 | CLL | 1.28 | 1.04-1.58 | 0.020 | 83.9% | 0.000 |
| Svenson 2009 | ccRCC | 1.35 | 1.10-1.65 | 0.004 | 83.1% | 0.000 |
| Svenson 2008 | breast cancer | 1.35 | 1.10-1.65 | 0.004 | 83.0% | 0.000 |
| Bechter 1998 | CLL | 1.27 | 1.03-1.56 | 0.022 | 83.7% | 0.000 |
| Kotsopoulos 2014 | ovarian cancer | 1.32 | 1.05-1.65 | 0.017 | 84.1% | 0.000 |
| Lin 2014 | bladder cancer | 1.30 | 1.05-1.61 | 0.014 | 84.2% | 0.000 |
| Shen 2012 | breast cancer | 1.33 | 1.07-1.64 | 0.009 | 83.5% | 0.000 |
| Hultdin 2003 | CLL | 1.28 | 1.04-1.57 | 0.022 | 83.7% | 0.000 |
| Gertler 2004 | colorectal cancer | 1.27 | 1.04-1.56 | 0.022 | 83.8% | 0.000 |
| Gertler 2008 | barrett carcinoma | 1.35 | 1.10-1.65 | 0.004 | 82.8% | 0.000 |
| Pezzolo 2015 | NB tumors | 1.35 | 1.10-1.64 | 0.004 | 83.0% | 0.000 |
| Chen 2015 | glioma | 1.30 | 1.05-1.61 | 0.017 | 84.0% | 0.000 |
| Qu 2015 | gastric cancer | 1.26 | 1.03-1.55 | 0.026 | 83.5% | 0.000 |
| Boscolo-Rizzo 2015 | HNSCC | 1.30 | 1.06-1.61 | 0.013 | 84.2% | 0.000 |
| Disease/Progression/Treatment free survival | | |  |  |  |  |
| Spanoudakis 2011 | MPN | 1.57 | 1.23-2.01 | 0.000 | 68.0% | 0.000 |
| Chen 2014 | colorectal cancer | 1.39 | 1.05-1.84 | 0.023 | 75.8% | 0.000 |
| Mansouri 2013 | CLL | 1.40 | 1.06-1.87 | 0.020 | 76.6% | 0.000 |
| Heaphy 2013 | prostate cancer | 1.39 | 1.05-1.84 | 0.020 | 76.3% | 0.000 |
| Jeon 2014 | NSCLC | 1.43 | 1.08-1.89 | 0.013 | 76.8% | 0.000 |
| Lu 2011 | breast cancer | 1.44 | 1.09-1.93 | 0.011 | 76.4% | 0.000 |
| Rossi 2009-1 | CLL | 1.40 | 1.05-1.86 | 0.023 | 76.3% | 0.000 |
| Rossi 2009-2 | CLL | 1.39 | 1.05-1.85 | 0.022 | 76.3% | 0.000 |
| Rampazzo 2012 | CLL | 1.37 | 1.05-1.81 | 0.023 | 74.7% | 0.000 |
| Borssén 2011 | CLL | 1.52 | 1.17-1.97 | 0.002 | 74.1% | 0.000 |
| Yan 2013 | AML | 1.39 | 1.05-1.84 | 0.021 | 76.0% | 0.000 |
| Roos 2008 | CLL | 1.43 | 1.07-1.90 | 0.015 | 76.8% | 0.000 |
| Garcia-Aranda 2006 | colorectal cancer | 1.50 | 1.16-1.95 | 0.002 | 74.1% | 0.000 |
| Pezzolo 2015 | NB tumors | 1.57 | 1.23-2.00 | 0.000 | 70.1% | 0.000 |
| Augustine 2015 | colorectal cancer | 1.41 | 1.06-1.87 | 0.017 | 76.8% | 0.000 |
| Chen 2015 | glioma | 1.42 | 1.05-1.92 | 0.022 | 76.6% | 0.000 |
| Qu 2015 | gastric cancer | 1.39 | 1.05-1.83 | 0.020 | 76.0% | 0.000 |
| Boscolo-Rizzo 2015 | HNSCC | 1.45 | 1.10-1.92 | 0.009 | 76.6% | 0.000 |

Abbreviations: ESCC: esophageal squamous cell carcinoma, CLL: chronic lymphocytic leukemia, NSCLC: non-small-cell lung cancer, HCC: hepatocellular carcinoma, ccRCC: clear cell renal cell carcinoma, MPN: myeloproliferative neoplasms, AML: acute myelocytic leukemia, NB: neuroblastoma, HNSCC: head and neck squamous cell carcinoma.
